# Book Reviews

**Published:** 1810-01

**Authors:** 


### CRITICAL ANALYSIS

OF THE

### RECENT PUBLICATIONS

ON THE

### DIFFERENT BRANCHES OF PHYSIC, SURGERY, AND MEDICAL PHILOSOPHY.

---

### *A Treatise on Champignons.* By M. PAULET, M. D.

[NOT having been able to procure this invaluable work, we offer the following Report, presented by Desfontaines to the National Institute.]

The work in question is divided into two parts. In the first, the author gives a methodical analysis of every thing that has been published on the subject of champignons, from the time of Theophrastus to the present day: nor do we recollect any writer that has escaped his notice. To this part are added critical observations on the nomenclature, synonomy and the salutary or deleterious qualities ascribed to certain species of champignons.

Among those whose writings stand in the highest estimation in this branch of science, M. Paulet distinguishes L'Ecluse, Jean Bauhin, Ray, Micheli, Tournefort, Vaillant, Linnæus, Haller, Schœffer, Bulliard, Batsch, and M. Persoon; the last of whom published a new classification of champignons a few years ago.

The second part of the work is divided into two chapters. The first containing general observations, and the second the description of the species; classed and arranged in an appropriate manner.

Champignons are described as pulpy, fleshy, coriaceous; sometimes resembling cotton, sometimes woody, or like cork, without leaves, petals, stamina, or pistils. On chemical analysis, their principles are analogous to those of animal substances; but they seem to be vegetables by their manner of growing, their reproduction, the places where they vegetate; and, finally, by their internal organisation. According to M. Paulet, they have a great affinity to the algæ; and, in his opinion, they form the last link which joins the animal to the vegetable kingdom.

Champignons grow in most abundance in temperate climates, and in woody countries. It appears that Europe produces them in greater plenty, than any other quarter of the globe. The most numerous species have their stalks terminated by an horizontal top, resembling a parasol; others are surmounted with a skin, plaited in different directions. Several have a bare stalk, and some have a globular form; such as the lycoperdons, or furze balls (*vesses de coup*).

In taste, smell, and colour, they differ considerably from each other.

other. A great number decay very soon; others continue longer on their stalks. Several are saturated with water, or filled with sap of various colours, and of different properties.

Champignons, when transplanted, never succeed. They perish in an instant; and great numbers are reduced to a black liquor, or rather into a fluid, resembling a *bouillie* of a fetid smell.

The soils and foreign bodies on which they grow, have a considerable influence on their consistence; but M. Paulet asserts very positively, in opposition to an opinion generally adopted, that the soil and climate do not, in the least, affect their poisonous or salutary effects.

The reproduction of champignons has long been a problem. Modern authors have discovered seeds in them resembling dust, and which are placed either on laminæ or appendices in particular boxes, or rather in the interior of the champignon itself; the skin of which is torn, in order to let them pass out. Their form is round or oval, and they are frequently projected out, as if by a spring. These seeds are connected together in a little net, as in the lycoperdons; or by a glutinous substance, as in what is called the white of the mushroom. They fall on the grass, or on the ground; and the author thinks that they are not altered when taken into the stomachs of animals, but are voided with the excrements in a round state.

We may easily see these seeds by suspending a champignon, in autumn, close to a mirror. The surface of the glass is soon tarnished and covered with a dust, which is the seed. We must here take the liberty to remark, that this opinion respecting the seed of champignons, does not seem completely unobjectionable.

M. Paulet assures us, that the white of the champignon, before germinating, is heated and swelled, and undergoes a perceptible fermentation.

The two chief causes of the developement of champignons, are heat and humidity combined: spring and autumn are the two seasons in which these requisites are prevalent in France. If summer and winter happen to be soft and rainy, they grow in these seasons also.

The most proper substances for favoring their developement, are vegetable matters in a state of decomposition; and animal matters combined with them, such as dung. When champignons grow on the trunks of trees, they indicate a decay in the wood.

The author afterwards treats of the signs, by which we may distinguish salutary from poisonous champignons; and points out the best method of counteracting the effects of those which are deleterious, when they have been taken into the stomach.

Almost all those of good quality are of a white, dry, firm texture, and grow in open places exposed to the sun. Those which are deleterious, grow under the shade of woods; they are less compact, most of them indeed are of a soft consistence; their surface is humid, and often viscous. Worms, snails, and other

 animals, animals, scarcely ever attack any champignons, but those which are salutary to human beings. Those may be regarded as suspicious that are heavy, and of a variegated colour externally; as also those which change colour when cut or broken. The same may be said of the species with bulbous stalks, proceeding from an envelope; and of those which grow at the foot of certain trees, particularly the olive, elder, yew, elm, or fig-trees. The autumnal months produce more poisonous champignons than the spring.

Champignons may be injurious in several different ways; in some cases, on account of their coriaceous texture; in others, on account of their cottony or spongy texture, which imbibe the juices of the stomach, and swells like a spunge; and in others, from their having undergone some alteration, as the common champignon, for instance, when it becomes black. The other kinds used, the lycoperdons, or furze-balls, when they have attained their state of maturity; i. e. when the pulp assumes a greyish colour, are equally prejudicial. Lastly, there are great numbers which contain a very deleterious resinous principle; such as the false orange, the green orange, &c. as they are called in France.

Several among the poisonous champignons do not produce their effects until ten or twelve hours after having been introduced into the stomach. These are the most dangerous of all; and the remedies which we may employ with success in other circumstances, are in these fruitless, and even frequently injurious.

From a great number of experiments made on animals and men, poisoned by champignons, M. Paulet asserts, that vitriolic ether sensibly diminishes the activity of the poison, and prolongs life a little; but that the most certain remedies are evacuants, particularly emetics, if given soon after the champignons have been received into the stomach. Oil, theriaca, milk, spirits, vinegar, and salt water, according to M. Paulet, have no effect. Oil and milk, however, may be employed as emollients, when the emetic has emptied the stomach.

After these general considerations, the author gives, in a second chapter, a new classification of champignons, according to their natural resemblance. He divides them into four Classes: the first comprehends those which have a hat or parasol, becoming thinner towards the edges. The second, those with a membranous skin, and of equal thickness throughout their whole extent; folded and plaited in different directions, like morels. In the third class are comprehended the fingered (digités) champignons, and those without hats. The fourth contains the globular champignons, the seeds of which are contained in the interior, such as the lycoperdons.

These classes are divided into orders, the orders into genera, and the genera into families, which comprehend the species. To these M. Paulet has given French names, and described them with great precision. To each of the descriptions are added numerous experiments made on animals, with a view to ascertain the deleterious effects ffects of various kinds of champignons; and to discover the best means of destroying or diminishing their influence. This part of the work is particularly interesting.

The author recounts a number of accidents from poisoned champignons in different countries. These species are much more numerous than is generally supposed. It would be very desirable to disseminate, as widely as possible, all the information we possess, with respect to the distinctive characters of good or bad champignons. In this respect the work is invaluable; particularly as it is accompanied by coloured engravings, extremely well executed. M. Paulet has been, from his infancy, attached to the study of this particular department in botany; and we trust that he will continue his inquiries on the subject.

---

*Facts establishing the Efficacy of the Opiate Friction in Spasmodic and Febrile Diseases. Also, Outlines of an Attempt to investigate the Nature, Causes, and Method of Cure, of Hydrophobia and Tetanus. Republished from the London Medical and Physical, and the Edinburgh Medical and Surgical, Journals. To which are added, Cases and Remarks, not before published.* By MICHAEL WARD, late Surgeon to the Manchester Infirmary, Dispensary, &c. 8vo. pp. 208.

THE candid Author of this valuable Production informs us, that he was first directed to try the effect of opiate frictions, by the account of a letter on the external use of opium, from Dr. Chiarugi, of Florence, contained in Duncan's Annals of Medicine for 1798; of which the following is a transcript:—

"In this letter the observations contained in the preceding article*, are partly contradicted and partly confirmed. The author, who is well known, from his very elaborate work on mental derangement, is chief Physician to the great Hospital of St. Boniface, which is appropriated entirely to maniacs. In treating their diseases, Dr. Chiarenti's discovery promised to be of great importance; and our author immediately resolved to carry it into practice. He was not, however, ignorant of the effects already observed by many practitioners from the external exhibition of this drug; and was persuaded that its action on the nervous system might be obtained, though not introduced into the stomach, without being dissolved in the gastric fluid, from the mere emanation of its volatile aroma. He, therefore, resolved to try it in the form of a simple ointment, made by incorporating a drachm of finely powdered opium with a pound of axunge. An ounce of

---

* "A Discourse on the Mode of acting on the Human Body, by means of Frictions made with Saliva and other Animal Fluids, and the various Substances commonly given internally. Recited in the University of Pavia. By Cit. V. L. Brera, M. D. Professor of Medicine, Clinical Lecturer and Surgeon to the National Legion of Pavia." 3d Edit. 1796.

this ointment, containing six grains of opium, procured sleep to a restless boy, who had been an ideot from his birth. Its effects were still more surprising on a peasant, in the heighth of a paroxysm of the most furious mania. *These frictions always rendered him calm, and sometimes threw him into a state of lethargic stupor. In this case a complete cure was at last effected.* It was exhibited, in the same manner, to twelve other persons, affected with mentalor re-active madness; and from his observations on them he concludes, that although the sleep produced was not always proportionate, either in intensity or duration, to the dose of the opium; *yet a state of calmness constantly succeeded, sooner or later; and five of them have been cured, without the use of any other remedy.* Certain of the efficacy of the opiate ointment, he tried frictions of laudanum, diluted with a little alcohol; and found them equally successful.

"These experiments being all made on patients who were tied, there could not be any suspicion that the opium was swallowed. Dr. Chiarugi thinks that they fully establish the fact, that opium, however applied to the skin, penetrates it; and exerts its action diffusively on the nervous system, without any necessity for its being mixed with gastric juice, provided it be sufficiently divided or diluted. Although, therefore, our author does not agree with Dr. Chiarenti in all his opinions, he confesses the utmost obligations to him, for having led him to a practice, from which he has derived much benefit in a disease, in which it is so difficult to exhibit medicines internally."

A considerable number of papers follow, extracted from our Journal, being communications from the Author concerning the efficacy of opiate frictions in nervous head-ach, chronic rheumatism, maniacal delirium, a delirium occurring under a fractured leg, chronic dysury, and calculus; typhus fever, cases of gangrene, symptomatic hiccough, case of vomiting and diarrhœa accompanied with hiccough; cholera morbus, hooping-cough, trismus traumaticus, &c.

After this, the Author enters on the subject of hydrophobia and tetanus. Some introductory remarks are premised, the leading articles of which have already also appeared in our Journal. The observations relative to the necessity of theory, and the authorities produced in its favor, are very judicious and unexceptionable. We trust, no one will dispute the absolute necessity of being somewhat prepared for an event so sudden, and hitherto so uncontrolable, as rabies canina. We transcribe the following passage, on account of the long note appended to it; which has not, we believe, hitherto appeared, but shows, at a single glance, the style, manner, and object of the Author:—

"*In hydrophobia, the spasmodic and retrograde motions are principally confined to the pharynx, the œsophagus, the larynx, the epiglottis, the tongue, and the muscles employed in deglutition;* (the stomach, though an involuntary muscle, is often affected in the same same manner); *hence the characteristic symptom of the disease, horror at the approach of liquids or food; hence also the inefficacy and fatal consequences of administering medicines internally, and the cruelty of urging the patient to swallow liquids. There are also convulsive motions of the heart and arteries, evinced by the violent palpitations which often take place. At the same time the voluntary muscles belonging to the chest and extremities are variously and violently agitated and convulsed* (the nervous power in them being abundant, and its action retrograde, but less so than in spasm; the energy of the brain seems also, in some cases, to be increased). *In some instances, there is merely an increased action of the voluntary muscles; in others, the latter are affected, partially or generally, with spasmodic or retrograde action, as in tetanus; all these circumstances contributing to produce that wonderful and horrible variety observable in the disease*.*"

On

* "If the delineation which is here given of the nature, causes, and phenomena, of hydrophobia, be in any tolerable degree correct, and I have taken great pains to render it so, we need look no farther for a solution of the long agitated question respecting *the uniform failure* of the treatment it has undergone, and the consequent mortality of the disease. The reason is, (as I have long since observed) "the plan which has been adopted is altogether improper." It is not to any particular medicine that I object, but to medicines generally, *given by the mouth.* The fact is, we have had recourse to means, or, what amounts to the same thing, *to methods of administering those means,* which it is impossible in the nature of things should ever succeed, *on account of the sensibility, irritability, and mobility of the pharynx and œsophagus, and the spasmodic and retrograde motions with which they are, in ninety-nine cases out of a hundred, affected.* Hence the necessity which I have long ago and repeatedly insisted upon, "Of an entire change in our manner of proceeding, before any progress can be made in the methodus medendi;" and the propriety "Of avoiding every thing which can tend to agitate and alarm, excite uneasy sensations, or bring on a return of the spasms." In conformity to this intention, instead of importuning the unfortunate sufferer to swallow medicines, or liquids, of which he has so great a dread, clysters should be given every four or five hours to support the strength, consisting of good broth, milk, &c. with from 30 to 40 drops of laudanum in each. And however long the present *irritating* plans of treatment may be continued, *to these, and others of a corresponding nature, we must, sooner or later, resort.* This, however, will, in all probability, be a work of time. Opinions of a contrary kind, which have been so long in use, do not immediately lose their influence. Besides, to propose to relinquish the *internal* use of medicines, and to substitute *external* remedies in their stead *(for it will be useless to compromise the matter by uniting the two plans, which seems to be the prevailing mode at present)*; and to call in question the propriety of the very common, but injudicious practice of inviting, *and even soliciting,* this pitiable class of patients to take medicines, drink water, &c. (which is infinitely more tantalising, and not more humane, than to propose a walk to a bed-ridden paralytic); or what is equally shocking to the feelings, of pouring water from one vessel to another in the same or an adjoining room, (when the *surprize* of the spectators is always in proportion to the

*horror*

On the supposition that diabetes is a spasmodic disease, the Author republishes his paper contained in our 7th volume, p. 503, to which we refer our Readers. Some other papers follow, extracted also from our Journal, containing further remarks on hydrophobia, tetanus, diabetes, and a case of opisthotonus successfully treated by Mr. Naylor of Gloucester. A case of trismus, cured by cold affusion, is also extracted from the Edinburgh Journal, some others from our own, and likewise from the publications of individuals.

A very interesting case of epilepsy is subjoined, in which the opiate frictions on the arms seem to have induced a temporary paralysis of those limbs. The patient continued to have slighter paroxysms for three succeeding years; "whether (says Mr. Ward) owing to the timely use of the proper means, or to a change in the habits of the patient, I cannot say certainly; probably to both of these causes." That the last is the most probable is to us confirmed by a subsequent Note, from which it appears that a change of diet, joined to a copious bleeding, had produced effects still more important.

"In an epileptic patient (says our Author) who was under my care so long ago as the year 1792, a permanent cure was effected by one copious bleeding during the paroxysm, followed after a short interval (which was employed in exhibiting an emetic, aperients,

---

*horror* expressed by the patient) are regulations so directly contrary to those which custom has established, that instead of being surprized at their not having produced all the effects I wish, the wonder is, all things considered, that they should have had any effect at all.

"But what renders the prospect still more discouraging is, that a formidable list of *internal* and other *remedies* (as they are called) still remains untried, each of which, it is to be feared, will be allowed its share of victims.

"Among others which have been proposed (exclusive of a multiplicity of *nostrums* which still retain their influence) are wine, either alone or mixed with some of the mineral acids or vinegar; thieves vinegar; wine and vinegar injected per anum; capsicum and other aromatics; some of the concrete acids, such as the essential salt of tartar, of lemons, or the flores benzoes, joined with capsicum or other aromatics, formed into bolusses with flour and water; ipecacuanha joined with acids and aromatics; tartarized antimony; copious bleeding, joined with an antiphlogistic regimen and medicines; nitrous and other mineral acids; olive oil in large quantities; strong purgatives; *cob-web;* &c. &c. And, as if to show how far, in this particular instance, credulity may be carried, *bronchotomy* has been advised by Dr. Rush! I am told too, that the experiment has been tried. Need I add, but without success.

"Besides these, there are others, such as cicuta, belladonna, and others of the narcotic tribe; pure caustic alkali; the arsenical solution; cantharides internally, and externally in liniments; the *mercurial* friction; electricity; galvanism; warm and cold *bathing*, &c. are strongly recommended as being deserving of a farther trial; but which should, I think, be banished from practice in the treatment of Hydrophobia, as being totally inadequate to the production of the effects expected from them, and therefore unworthy of the confidence which has been reposed in them.

rients, &c.) by a moderate use of cold bathing, and a complete change in the accustomed habits with regard to diet, exercise, &c."

The following case is scarcely less interesting. "M. J. H——, æt. 22, addicted tohard drinking, has had repeated attacks of maniacal delirium ; the last and most severe of which was in August 1808. After several very turbulent and sleepless nights, I was called in on the evening of the 30th. A considerable degree of fever prevailed ; the circulation was hurried ; *he talked incessantly*, and every movement was expressive of the greatest possible degree of terror and alarm ; to escape from the imaginary causes of which, he was constantly endeavouring to get away from his attendants, especially during the night.

"Bleeding, refrigerants, purgatives, antispasmodics, and the opiate friction, were prescribed ; a strait waistcoat was also procured, to be in readiness : a precaution which happily proved unnecessary.

"The opiate friction was applied soon after he had been bled, (both of which were performed with difficulty, owing to the extreme restlessness) and ordered to be repeated every two or three hours, till he became more composed ; the good effects of which were very soon evident, so much so, that it was used only twice in the course of the night : and at my next visit, the day following, I found him in a state of comparative ease and composure both of body and mind.

"Without continuing the narrative it will be sufficient to observe, that he was so completely recovered by the 5th of September, as to require no farther assistance, either medical or otherwise."

Another similar case is related by Dr. Bardsley. This success induced the author to make trial of the remedy in many other cases of spasm or convulsion, and often with advantage ; among others, in convulsions during the eruptive fever in small-pox. The mention of this disease produces a Note on cow-pox, and a wish for parliamentary interference relative to the variolous infection. We heartily join in the wish, that the question should be subjected to such an inquiry as may lead to a just knowledge of its whole bearing. We shall here conclude our remarks with thanking the Author for the evidence he has collected on the mode of exhibiting an important remedy, which seems likely to alleviate diseases hitherto considered as desperate.

---

*The Physician's Vade-Mecum: containing the Symptoms, Causes, Diagnosis, Prognosis, and Treatment of Diseases ; accompanied by a Select Collection of Formulæ, and a Glossary of Terms.* By Robert Hooper, M. D. &c. pp. 270. 8vo. London, 1809.

A considerable number of works have appeared very analogous to the present, under the titles of "*Outlines ; Synopses ; Text Books ; Medical Pocket Books ; Vade Mecums, and London Practices of Physic* ;

*sic*; the leading object of all being professedly, to exhibit a concise view of the state of the practice at the time they were published.

The general *theory* of diseases has long become pretty steady, or perhaps, a general indifference to it has discouraged speculative writers; but the *practice* is perpetually varying, on account of the introduction of new remedies, or new modes of exhibiting the old ones. A new disease occasionally presents itself also, which requires some time and experience to ascertain the best manner of treating it. Of this kind are the Yellow Fever, Cow pox, Tic Doloureux, &c. Several diseases also which have been long known, but the treatment of which is generally ambiguous or unsuccessful, will never fail to exercise the industry and ingenuity of practitioners. To this class belong, Hydrophobia; Hydrothorax; Gout; Chronic Rheumatism; Mania; Cancer, &c.

These causes are sufficient to stimulate experienced, or experimental practitioners, to communicate every improvement of importance to the public as soon as possible; and we rejoice in being able to say, that this Journal has been the vehicle of many such improvements.

The present publication, agreeably to the object of such works, exhibits an outline of the state of the *practice of medicine* at this time, according to the author's ideas on the subject. We must, however, inform our readers, that the names employed in the New London Pharmacopœia could not be introduced into this publication, as it was probably printed some time antecedent to the publication of that work. This defect may easily be supplied at present, by means of the lists, containing the new and old names, sold by all the medical booksellers; doubtless, the next edition will contain the new names.

In the execution of this work the author has followed the arrangement and names of Dr. Cullen's Nosology, which is now generally followed in these islands, and is, certainly, the best calculated for teaching. But in distinguishing some of the species, he has suffered himself to be too implicitly guided by that great authority, for the general purposes of practice.

As a Text-book for lectures, such specific distinctions may be very proper, but as a guide to young practitioners, we think them liable to mislead.

The distinction, for instance, between typhus mitior and gravior; scarlatina cynanchica, and cynanche maligna; sanguienous and serous apoplexy; with several others of this kind, must be considered only as land marks to the lecturers, not as guides to practice.

In the preface, the author says, "he has discarded all theory, and retained only those leading facts which it is absolutely necessary for a practitioner to be acquainted with, when he approaches the bed-side of a patient."

The order in which each disease is treated we think unexceptionable, able, and commences generally with the nosology. An enumeration of the symptoms and signs is next given, which very properly introduces an account of the causes both predisposing and exciting. The author has purposely, and we think very judiciously, avoided those useless theoretical disquisitions about proximate causes, which tend more to embarrass than promote the science of medicine.

The accurate distinction of diseases being a point of the greatest importance in practice. Dr. H. has given all the diagnostic signs and symptoms with particular care. The *prognosis* is often involved in considerable difficulty, and yet the relations and friends of the patient always expect the medical attendant to give an early opinion on that subject. The proper directions on this head are always given.

The *treatment*, or manner of conducting the cure, concludes each disease; and after the proper indications have been laid down, and the means of fulfilling them pointed out, we have a very copious list of remedies, both simple and compound.

Though the above exhibits the general plan of treating each malady, there must necessarily be many deviations from it. Several morbid affections have a *specific* exciting cause which requires no predisposition: some few have no known remedy, as hydrophobia and cancer; in which the prognosis also, is sufficiently obvious without written instructions.

As this work is designed solely for the use of those who are intended to practice as physicians, no mention is made of local or surgical complaints. We think it compiled with attention and judgment, well calculated to answer the purposes for which it was intended.

---

*Remarks on the Purulent Ophthalmy which has lately been Epidemical in this Country.* By JAMES WARE, Surgeon, F. R. S.

BY some accident this little tract was so long overlooked, that we should have left it unnoticed here, had it not been that the disease alluded to, still continues most seriously to pervade several regiments. However, Mr. Ware's object is to show that a purulent ophthalmy has been epidemical in England before the Egyptian expedition. Of the progress and symptoms of this disease, with a most accurate and correct account of the cause, we shall offer Mr. Ware's opinion in his own words.

"It is difficult to discover in what way this disorder was first occasioned. The resemblance which it bears to that species of ophthalmy, which in many instances, has either accompanied or followed the common gonorrhœa, strongly impresses my mind with an idea, that the two disorders bear a close reference one to the other. I believe it is admitted by the most experienced surgeons, that the gonorrhœa, in by far the greater number of instances, is perfectly distinct from the lues venerea; and that the remedies which are indispensibly required for the cure of the latter, are wholly unnecessary for the cure of the former The first cause of the gonorrhœa we do not know; but it is communicated by the application of a peculiar poison to the urethra, which inflames and excites a considerable purulent discharge from it. It is rarely productive, however, of any ulceration in the inflamed part, or any affection of the general constitution. In like manner, the purulent ophthalmy, (without inquiring at present into its first cause) appears to me to be in general communicated by the application of a peculiar poison to the tunica conjunctiva, which inflames and produces a considerable discharge from it, but rarely occasions any ulceration in this tunic,* or any affection of the general system. Infants, as well as adults, are subject to the purulent ophthalmy: and it is a fact, well deserving notice, that some of the worst cases of this disorder that have occurred in infants, have happened in those whose mothers were subject to an acrimonious discharge from the vagina at the time the infants were born. Some of the worst cases also, that have occurred in adults, have happened in those, who either shortly before the attack of the ophthalmy, or at that very time, laboured under either a gonorrhœa, or gleet.† I do not mean to attribute every opthalmy of this kind to such a cause. I am aware that it has sometimes occurred, and in the most violent degree, when no such circumstance could be suspected; but in the far greater number of adults whom I have seen affected by it, if the disorder had not been produced by the application of morbid matter from a diseased eye, I have been able to trace a connection between the ophthalmy and some degree of morbid affection in the urinary canal."

Mr. Ware proceeds further to remark, that this purulent ophthalmia, even when arising from the contact of gonorrhœal matter, is not to be confounded with the true venereal ophthalmia occuring among the secondary symptoms of that disease, and which he describes thus.

"In cases of this latter description there is generally a great exacerbation of pain during the night, and the internal parts of the eye are particularly affected. The pupil is usually contracted, and loses the power of altering its size in different degrees of light. The iris

* Strictures in the urethra, not unfrequently follow a gonorrhœa; and ulcers of the cornea, as well as a rupture of this tunic, not unfrequently follow a purulent ophthalmy. But the former does not afford a proof of the previous existence of an ulcer in the urethra; nor does the latter, that an ulceration had previously taken place in the tunica conjunctiva.

† "Dr. Vetch observes in his account of "the Ophthalmia which appeared in England since the return of the British army from Egypt," p. 3, that in the 2d battalion of the 52d regiment, in which the disorder occurred, with a severity then unprecedented in this country, that "*excepting a great proportion of venereal cases*," no particular distemper seemed to prevail; and the number of patients in the hospital were rapidly decreasing, when the first case of ophthalmia made its appearance. Among these venereal cases is it not highly probable that not a few had the gonorrhœa?

iris assumes a greenish colour, and pustules not unfrequently form upon it."

We cannot help suspecting some ambiguity in this account, though we are ready with the author to admit that the purulent ophthalmia, if venereal, must in all probability arise from the immediate cause to which he ascribes it, because we have no instance in which secondary symptoms of lues venera affected mucous membranes, excepting by ulceration.

Abstracted from the practical remarks, the above are the principal objects of this useful little pamphlet. The practical remarks, though highly valuable, as might be expected from the long experience of the author, are so compressed, that we must refer those who feel particularly interested in this branch of the profession to the work itself.

---

*Identities Ascertained; or an Illustration of Mr. Ware's Opinion respecting the Sameness of Infection in Venereal Gonorrhœa, and the Ophthalmy of Egypt; with an Examination of Affinity between ancient Leprosy and Lues.*

In commenting on Mr. Ware's opinion, the lively author of this little production seems alternately in joke and in earnest. We find it extremely difficult to conceive him in earnest, when he admits, with so much ease, a position of Mr. Jesse Foot, and drawing an inference from it, which, even if the position were just, would be neither necessary nor even consequent.

"If, says he, the purulent opththalmia does aries from the contact of gonorrhœal matter, how are we to explain the reason why the two diseases so rarely appear together?" As if it were necessary, or even common for men with gonorrhœa, to bring any of the discharge into contact with their eyes. If they were likely, by such mean, to infect any mucous membrane, distant from the urethra, we should think it much more probable to be the nostril. To us, therefore, there appears no difficulty in assigning a reason why the two diseases should not more generally appear together; least of all, can we admit the explanation from the unfounded assertions alluded to.

"The principle to which allusion," says our author, is now made, was disclosed by Mr. Foot, in a short publication, under the title of his *newly discovered Fact*, some years ago; and consisted in this position, that the matters of gonorrhœa or chancre are not capable of producing any effect on any other part of the body *of that individual from whom they are derived.* The animal body cannot contract, in a new situation, these poisons, from matter yielded by itself. When there are profuse discharges from virulent gonorrhœa or corroding chancres, the application of matter must be made, in almost every instance, to various parts of the body, and even to surfaces as susceptible of being acted upon as those of the *pudenda* of either sex. The *tunica conjunctiva* of the eyes eyes is equally susceptible—the lips are susceptible—the nipples of a woman are susceptible—the anus is susceptible. But these diseases are never communicated to these parts from any person's own body. A chancre in the glans penis is never communicated to the præpuce, where there is a perpetual point of contact. It was supposed that contiguity of situation rendered parts unsusceptible. This was endeavouring to account, in a very superficial way, for what could not be comprehended: for it had never been presumed, that every part of the identical body was equally unsusceptible as parts in the neighbourhood. It was a limited assent to that principle, which is general over the body—an acknowledgment extorted from observation of matter being in constant application to the neighbouring parts without any effect being produced upon them. The inability, however, of receiving impression from the poison, does not depend on contiguity or remoteness, but is universal over the surface. When this is admitted, there will be no difficulty in conceiving the matters of gonorrhœa and of purulent ophthalmy to be the same, although they are so seldom known to have occurred together in the same subject."

Is it not well known, that Mr. Hunter inoculated two subjects with their own venereal matter, and produced chancres? That the Suttons and Dr. Woodville inoculated subjects from their own primary variolous pustules, and thus contrived to satisfy their minds by the appearance of a general eruption? Need we mention the number of instancess recorded in our Journal, of young subjects who have infected themselves by their own vaccine virus?

We agree with the author, in considering the idea of metastasis, by some called the retrocedent gonorrhœa, as a wild hypothesis, and that if a purulent ophthalmia ever arises from such a cause, it must be by actual contact; but again, we see no reason why that matter should not be from the same individual. It is further urged, that if such were the cause, we should sometimes find ulcers in the eye, as gonorrhœal matter may produce chancres. It should however be recollected, that the eye is a secreting surface as well as the urethra, and therefore less likely to be ulcerated; but it is not less certain, that ulceration does sometimes occur, probably quite as often at the comparative ratio between the gonorrhœa and ulcers at the mouth of the urethra. This part of the argument is concluded by a reference to the controversy which has so often come before us, concerning the identity of the venereal and chancrous virus. As the author refers to no experiments, it is not to be wondered if he leaves the matter undecided, though he seems to lean to the opinion that the virus of each is different.

This leads to another suggestion, concerning the existence of the venereal disease, under the name of leprosy, anterior to the siege of Naples. In this, the old controversy of Becket, Astruc, and others, is renewed; and at last, the author, not content with the identities before remarked, seems determined to confound all distinctions.

"In

"In recent times, the primary affections of the venereal disease were in possession of the mouth in some parts of the world, and the communication of infection the same as in leprosy of old. An ingenious pamphlet was published about thirty years ago, respecting sunch a condition of the disease in Paul's Head Bay, on the Northern coast of America. The name of the author does not now recur to the mind. A disease, under similar shape, has been long known in Scotland, among people of the lowest condition, under the denomination of Sibbens. Mr. Benjamin Bell has made particular mention of it in his Treatise on the Venereal Disease. There is not a doubt that these diseases were, in both instances, the true venereal malady, with all that acrimony of contagion which distinguishes a primary venereal ulcer wherever it may be found. They were genuine leprosy, of which the circulation did not take place by venereal intercourse, but from other opportunities of contact in the common concerns of life; and if the aid of mercury was not cultivated, would have proceeded to as dreadful extremity as leprosy in any age. They have been considered a different kind of the venereal disease. The only difference is in the primary symptoms, which alone can convey infection, being in a different situation. Upon the surface of a spoon, or the edge of a drinking cup, their matter would communicate the most malignant venom to the lips or fauces of another person, while perfectly innocent to every other part of the surface of the body by which it was produced."

To say no more, it is at least bold in this anonymous Writer, to offer so decided an opinion on three diseases, two of which he does not pretend ever to have seen, yet modestly sets to rights those who have taken pains to mark all the distinctions.

The remainder of the pamphlet again refers to the strange notion, that matter from the same individual, applied to a different part, will produce no infection. On this subject, we wish the Author to study Mr. Hunter, instead of skimming over the pages of Mr. Foot.

